# Conventional and genetic risk factors for chronic Hepatitis B virus infection in a community-based study of 0.5 million Chinese adults

**DOI:** 10.1038/s41598-022-16360-7

**Published:** 2022-07-15

**Authors:** Elizabeth Hamilton, Ling Yang, Alexander J. Mentzer, Yu Guo, Yiping Chen, Jun Lv, Robert Fletcher, Neil Wright, Kuang Lin, Robin Walters, Christiana Kartsonaki, Yingcai Yang, Sushila Burgess, Sam Sansome, Liming Li, Iona Y. Millwood, Zhengming Chen

**Affiliations:** 1 Clinical Trial Service Unit and Epidemiological Studies Unit (CTSU), Nuffield Department of Population Health, BDI Building, Old Road Campus, University of Oxford, Oxford, OX3 7LF UK; 2 Medical Research Council Population Health Research Unit (MRC PHRU), Nuffield Department of Population Health, University of Oxford, Oxford, UK; 3 The Wellcome Centre for Human Genetics, University of Oxford, Oxford, UK; 4 Chinese Academy of Medical Sciences, Beijing, China; 5 Department of Epidemiology and Biostatistics, School of Public Health, Peking University Health Science Center, Beijing, China; 6 The George Institute for Global Health, Sydney, Australia; 7 NCDs Prevention and Control Department, Shinan CDC, Qingdao, Shandong China; 8 Center for Public Health and Epidemic Preparedness and Response, Peking University, Beijing, China

**Keywords:** Virology, Infectious diseases, Liver diseases, Hepatitis, Liver cancer, Liver cirrhosis, Microbiology, Risk factors

## Abstract

Despite universal vaccination of newborns, the prevalence of chronic hepatitis virus B (HBV) infection and the associated disease burden remain high among adults in China. We investigated risk factors for chronic HBV infection in a community-based study of 512,726 individuals aged 30–79 years recruited from ten diverse areas during 2004–2008. Multivariable logistic regression was used to estimate odds ratios (ORs) of hepatitis B surface antigen (HBsAg) positivity recorded at baseline by sociodemographic and lifestyle factors, and medical history. In a random subset (n = 69,898) we further assessed the association of 18 single nucleotide polymorphisms (SNPs) previously shown to be associated with HBsAg positivity and development of chronic liver disease (CLD) (1600 cases). Several factors showed strong associations with HBsAg positivity, particularly younger age (< 40 vs. ≥ 60 years: OR 1.48, 95% CI 1.32–1.66), male sex (1.40, 1.34–1.46) and urban residency (1.55, 1.47–1.62). Of the 18 SNPs selected, 17 were associated with HBsAg positivity, and 14 with CLD, with SNPs near *HLA-DPB1* were most strongly associated with both outcomes. In Chinese adults a range of genetic and non-genetic factors were associated with chronic HBV infection and CLD, which can inform targeted screening to help prevent disease progression.

## Introduction

Hepatitis B virus (HBV) infection is one of the most common chronic viral infections in the world, affecting 240 million people globally^1^ and accounting for 1.3 million deaths each year mostly from cirrhosis and hepatocellular carcinoma (HCC)^1^. China accounts for one-third of all global chronic HBV cases^1^, despite widespread and free universal vaccination of newborns since 2005^2^. The prevalence of chronic HBV infection among middle-aged to older adults in China remains relatively high^3^, and without treatment up to one-quarter of those infected ultimately develop cirrhosis or liver cancer^4,5^. There is ongoing high mortality associated with chronic HBV, with an estimated 10 million people living with chronic HBV in China predicted to die by 2030 from liver cancer and chronic liver diseases^6^.

Improving the identification of chronically infected individuals is a key component to addressing the chronic HBV burden in China^6^. It is described as a ‘silent epidemic’^1^ reflecting the asymptomatic disease course that contributes to late diagnosis and poor prognosis – in China the diagnosis rate is estimated at less than 20%, well below the World Health Organization (WHO) target of 90% by 2030^7,8^. However, apart from risk factors contributing to mother to child transmission, comparatively little is known about risk factors for HBV chronicity in adults, and the most recent nationwide Hepatitis B serosurvey in China in 2014 only included participants aged 1–29 years^9^. Furthermore, in addition to conventional risk factors, genome wide association studies (GWAS) have found several genetic variants associated with chronic HBV. Most variants are human leukocyte antigen (HLA) loci, which play a critical role in the host immune response to viral infection through antigen presentation^10^, where polymorphisms can alter the efficacy of antigen binding and T-cell response, impacting viral clearance^11^. Other genes (including some in non-HLA regions of the genome) also impact likelihood of viral persistence or clearance by altering the magnitude of adaptive or innate immune responses^11^. Existing GWAS were relatively small case–control studies in constrained geographical areas, recruiting cases from hospitals or liver cancer screening units^12–16^, and further research examining how these genetic variants are related to chronic HBV risk in a large, geographically diverse population sample is of interest.

Further knowledge about risk factors associated with HBV chronicity in adults may help ongoing efforts to reduce the chronic HBV burden in China, by informing targeted testing of higher risk individuals to capture infected individuals on the chronic HBV care continuum, to receive appropriate treatment and care^17,18^. We used a large community-based cohort study of middle-aged adults from ten geographically diverse sites in China to assess both genetic and non-genetic risk factors associated with chronic HBV.

## Methods

### Study population

The China Kadoorie Biobank (CKB) study design has been described in detail elsewhere^19^. A baseline survey was conducted in 2004–2008 among 512,726 men and women, aged 30–79 years, recruited from five urban and five rural geographically diverse areas in China. Potentially eligible participants were identified through official residential records in each of 100–150 administrative units (rural villages and urban residential committees) within each region. Trained health workers administered laptop-based questionnaires at local study clinics collecting information on sociodemographic and lifestyle factors (e.g. smoking, alcohol consumption, diet, physical activity) and medical history (e.g. history of blood transfusion, self-reported health and medical conditions diagnosed by a doctor including whether they had a history of chronic hepatitis or cirrhosis). Blood pressure, lung function and anthropometric measures were measured using standard protocols, and a non-fasting venous blood sample was collected at baseline for on-site tests and long-term storage. Resurveys following similar procedures were conducted in 2008 and 2013–2014 among a subset (4–5%) of surviving participants. Vital status of participants was determined periodically through national death registries, and episodes of hospitalization were collected via linkage to disease registries and national health insurance claims database, which has almost universal coverage in study areas. International Classification of Diseases, 10^th^ Revision (ICD-10) were used to code disease events. Prior international, national and regional ethics approvals were obtained and all participants provided written informed consent.

### Measurement of chronic HBV infection

Hepatitis B surface antigen (HBsAg) was measured in all participants at the baseline visit, using a point of care, lateral flow rapid diagnostic test (RDT), where participants’ venous whole blood was applied to an on-site rapid test strip (ACON dipstick). Results were recorded as positive, negative, or unclear. HBV antibodies to hepatitis B core antigen (anti-HBc) and hepatitis B e antigen (anti-HBe) were additionally measured in stored plasma samples from a randomly selected subcohort of 2000 participants who were alive and cancer free after two years of follow-up, using a Luminex-based multiplex serology panel^20^.

### Genotyping and genetic variant selection

The present study used a subset of 75,982 genotyped samples using a custom-designed 800K-SNP array (Axiom; Affymetrix). This sample was approximately representative of the overall CKB cohort, where selection was by box of DNA samples, prioritising individuals from second resurvey study clinics (which were representative of the cohort) or at random from other recruitment sites. After exclusion of 4945 individuals who were regional population outliers based on genomic principal components analysis within regions, there were 71,037 people in the genetic analyses of chronic liver disease (CLD), and after further exclusion of 1139 participants with missing HBsAg data, there were 69,898 participants included in the main genetic analyses for HBsAg positivity (Supplementary Fig. 1). Replication of genetic associations were performed on 18 SNPs previously found to be associated with chronic HBV infection in prior GWAS. These were identified by searching the US National Human Genome Research Institute Catalog of Published GWAS^21^ for “hepatitis B virus infection trait” (Trait: EFO_0004197, searched August 2020) and limiting findings to GWAS reporting SNPs associated with persistent HBV infection, or susceptibility to HBV infection reported, and where the finding had been replicated (either in the relevant study or subsequent GWAS). Studies^10,12–14,16,22–24^ reporting these SNPs are summarized in Supplementary Table 1–2.

### Disease outcomes in genetic analyses

CLD was defined as participants with either prevalent or incident liver disease. Prevalent liver disease included participants reporting either cirrhosis/chronic hepatitis or liver cancer diagnosed by a doctor in the baseline survey. Incident liver disease was captured by electronic health record linkage described above, including cirrhosis [ICD10: K70, K74], hepatic failure [K72] or liver cancer [C22]). A total of 1600 people (2.3%) had CLD at baseline or occurring during the follow-up out of 71,037 participants in the GWAS randomly selected sample (Supplementary Fig. 1).

### Statistical analysis

Individuals with missing body mass index (BMI) (n = 2) or missing/unclear HBsAg (n = 11,733) data were excluded, leaving 500,991 participants for the main analysis. Prevalence estimates were generated for HBsAg and HBV antibodies by baseline characteristics, standardized by age (10-year categories) and study site among men and women separately. Logistic regression models were used to estimate odds ratios (OR) and 95% confidence intervals (CI) for HBsAg positivity associated with a range of baseline characteristics, including demographic, socioeconomic, behavioural and medical risk factors. In the basic model, we adjusted for age (5-year categories), sex and study site (10 areas). A forward selection method was then used to determine which factors were included in the multivariable model, where the likelihood ratio test (LRT) was used to compare the basic model with sequential addition of risk factors and those that significantly improved model fit were retained in the model (Supplementary: Table 3, 4 and Methods S1 for details). Given the high proportion of participants that consumed regular vegetables and were currently married (all > 90%), these factors were not included in multivariable analysis. Tests of trend were performed across ordered categorical variables.

Genetic associations were analysed using an additive model for individual SNPs. SNPs were orientated so that the risk allele was the allele associated with HBsAg positivity in existing GWAS. Logistic regression models for HBsAg status (positive, negative) and chronic liver disease (CLD, yes vs. no) were fitted, stratified by ten study sites and adjusted for age (10-year categories), sex and up to ten region specific principal components. Inverse-weighted fixed effect meta-analyses were used to calculate overall estimates and 95% CI. Additional analyses were conducted to investigate risk of progression to CLD among HBsAg positive participants, and, in the subset of 2000 people with HBV antibody data, the risk of HBsAg positivity among those exposed to chronic HBV, as measured by anti-HBc status).

All analyses used R v4.0.2 and PLINK v2.0^25^. We considered two-tailed *p* < 0.05 as evidence of an association. To account for multiple testing in the genetic analyses we applied a Bonferroni correction to the significance level, dividing 0.05 by 18 SNPs tested (i.e., 0.003).

### Role of the funding source

The funders of the study had no role in study design, collection, analysis or interpretation of data, or in the writing of the report.

### Ethical approval and informed consent

The China Kadoorie Biobank (CKB) complies with all the required ethical standards for medical research on human subjects. Ethical approvals were granted and have been maintained by the relevant institutional ethical research committees in the UK and China: UK – Kadoorie Study of Chronic disease in China (KSCDC) - Baseline, Oxford Tropical Research Ethics Committee (OxTREC) (2005) OxTREC Ref: 25-04; China– Kadoorie Study of Chronic disease in China (KSCDC) – Baseline, Chinese Centre for Disease Control and Prevention. Ethical Review Committee (2004) Approval Notice 005/2004. Informed consent was obtained from all participants included in the study.

## Results

### Baseline characteristics and prevalence of chronic HBV infection

**Figure 1:**
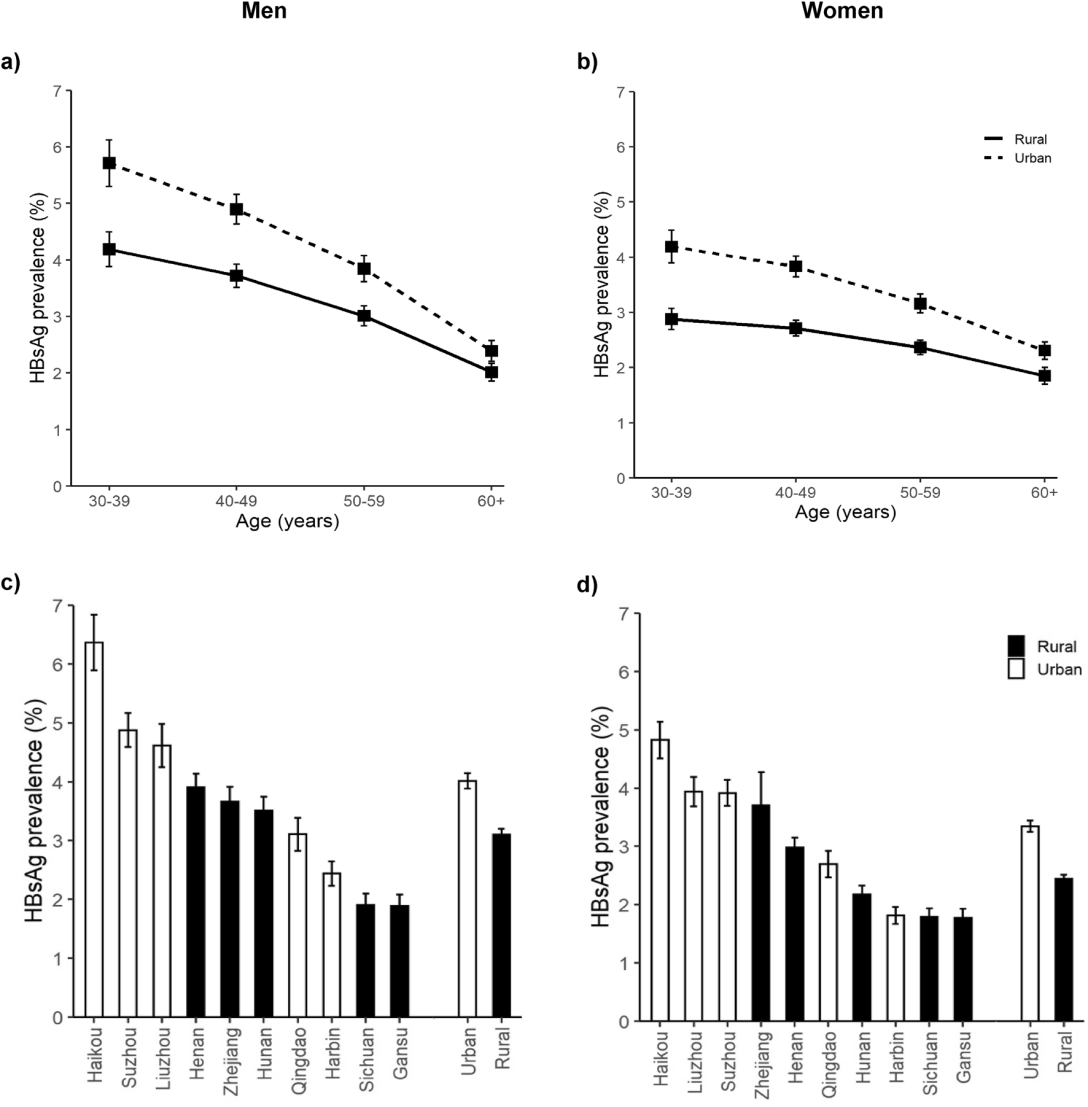
Prevalence of Hepatitis B surface antigen by age, sex and study area. Hepatitis B surface antigen (HBsAg) prevalences displayed (95% CI) are standardized by age (10-year categories) and study site (ten sites) among men and women separately, stratified by urban or rural location. (**a**) HBsAg prevalence in men by age category, (**b**) HBsAg prevalence in women by age category, (**c**) HBsAg prevalence in men by study site, (**d**) HBsAg prevalence in women by study site. HBsAg, hepatitis B surface antigen.

Among the 500,991 participants included, the mean (SD) age was 52.1 (10.7) years, 41.0% were men, 55.4% lived in a rural area, 18.2% had attained primary or middle school education. The overall HBsAg prevalence was 3.0% (n = 15,552), which was higher in men (3.4%) than in women (2.8%). HBsAg prevalence decreased with age particularly in men (Fig. 1A,B), and varied across study areas (Fig. 1C,D), with the highest prevalence in Southern Haikou (women: 4.8%; men: 6.4%) and lowest in Western Gansu (women: 1.8%; men: 1.9%). Overall, urban sites had a higher prevalence than rural sites among women (3.3% vs. 2.4%) and among men (4.0% vs. 3.1%;). HBsAg prevalence was higher in those less educated, agricultural workers and those with lower household income, while prevalence decreased across increasing number of years with a household fridge (Supplementary Fig. 2). Of HBsAg positive participants, 11.3% reported chronic hepatitis or cirrhosis at baseline, of whom 18.9% were on current treatment (Table 1). In the subcohort with HBV antibodies measured, overall seroprevalence was 45.0% and 44.8% for anti-HBc and anti-HBe respectively, where seropositivity for both antibodies were higher in males than females, and increased with older age (Supplementary Table 5).

**Table 1:** Baseline characteristics of overall cohort and by Hepatitis B surface antigen status.

	HBsAg Negative (N = 485,439)	HBsAg Positive (N = 15,552)	Overall (N = 500,991)
Age (years), mean (SD)	52.1 (10.7)	49.8 (10.0)	52.1 (10.7)
Men, %	40.8	46.1	41.0
Rural residence, %	55.6	48.8	55.4
Married^a^, %	90.5	91.8	90.6
No formal education, %	18.3	15.6	18.2
Agricultural occupation, %	41.3	36.9	41.2
Household income ≥ 20,000 Yuan, %	42.6	46.5	42.7
Household size ≥ 4 people, %	51.9	53.0	52.0
No household fridge, %	45.4	43.2	45.3
**Current smoker**^**b**^**, %**			
Men	72.2	73.8	72.2
Women	4.2	3.0	4.1
**Ever-regular alcohol intake**^**c**^**, %**			
Men	48.1	46.9	48.1
Women	4.3	3.4	4.3
**Dietary factors, regular intake**^**d**^**, %**			
Vegetable intake	98.3	98.5	98.3
Fruit intake	28.1	27.5	28.1
Physical activity (MET-hours/day), mean (SD)	20.9 (13.8)	21.9 (14.3)	21.0 (13.8)
SBP (mmHg), mean (SD)	131 (21.3)	129 (20.4)	131 (21.3)
BMI (kg/m^2^), mean (SD)	23.7 (3.4)	23.5 (3.4)	23.7 (3.4)
Blood transfusion, %	4.3	4.9	4.3
Poor self-rated health, %	10.3	11.0	10.3
**Prior disease history, %**			
Chronic HBV/cirrhosis	0.9	11.3	1.2
Current treatment	10.4	18.9	12.9
Cancer	0.5	0.6	0.5
CHD	3.1	1.8	3.1
Stroke or TIA	1.8	1.3	1.7
Diabetes	3.2	2.6	3.2

HBsAg, hepatitis B surface antigen; MET, metabolic equivalent of task; SBP, systolic blood pressure; BMI, body mass index; CHD, coronary heart disease; TIA, transient ischaemic attack.

^**a**^Married: Participants who reported currently being married, ^**b**^Smoking: Current smoker includes those reporting occasional or current smoking, ^**c**^Alcohol: Ever-regular alcohol intake includes participants reporting monthly, reduced intake, weekly or ex-regular alcohol intake, ^d^Dietary factors: regular includes participants report intake 4 or more times per week.

### Conventional risk factors associated with HBsAg positivity

Table 2 shows relationships between a range of conventional risk factors measured at baseline with HBsAg positivity. Compared to their counterparts, participants who were of younger age, male, resident in urban sites, underweight, with no formal education, with a history of blood transfusion or with poor self-reported health status at baseline had higher HBsAg positivity; while the converse was true for people with higher household income, occasional alcohol intake, longer use of a household fridge and who were overweight (all *p* < 0.001). Of these, the strongest associations were seen with age (< 40 vs. ≥ 60 years old: OR 1.48 [95% CI 1.32–1.66]), sex (male vs. female: 1.40 [1.34–1.46]), study area (urban vs. rural: 1.55 [1.47–1.62]) and self-rated health (poor vs. good health status: 1.29 [1.22–1.37]). Compared to non-drinkers, occasional and ever-regular alcohol drinkers had lower HBsAg positivity. For BMI, compared to the normal BMI category, underweight participants had higher HBsAg positivity (1.11, 1.03–1.20), while the opposite was true for overweight participants (0.92, 0.89–0.95). Occupation, household size, physical activity, smoking and fruit intake were not associated with HBsAg positivity after multivariable adjustment (Table 2).

**Table 2:** Odds ratios and 95% CI for baseline factors by Hepatitis B surface antigen status.

	N cases	Model 1^a^		Model 2^b^	
		OR (95% CI)	*P*-value^c^	OR (95% CI)	*P*-value^c^
**Age, years**					
≥ 60	2611	*Ref*	< 0.001	*Ref*	< 0.001
50–59	4587	1.44 (1.37–1.51)		1.14 (1.05–1.24)	
40–49	5390	1.80 (1.71–1.88)		1.33 (1.20–1.47)	
< 40	2964	2.03 (1.93–2.15)		1.48 (1.32–1.66)	
**Gender**					
Men vs. women	7176	1.28 (1.24–1.32)	< 0.001	1.40 (1.34–1.46)	< 0.001
**Study area**					
Urban vs. rural	7962	1.35 (1.31–1.40)	< 0.001	1.55 (1.47–1.62)	< 0.001
**Birth cohort**					
< 1940	1146	*Ref*	< 0.001	*Ref*	< 0.001
1940–49	2573	1.15 (1.02–1.30)		1.28 (1.18–1.39)	
1950–59	5175	1.32 (1.13–1.54)		1.64 (1.48–1.83)	
1960 onward	6658	1.37 (1.15–1.65)		1.75 (1.55–1.98)	
**Education**					
High school or above	3373	*Ref*	< 0.001	*Ref*	< 0.001
Primary or middle school	9746	1.16 (1.11–1.21)		1.08 (1.03–1.13)	
No formal education	2433	1.25 (1.17–1.34)		1.15 (1.07–1.23)	
**Occupation**					
Agricultural	5738	*Ref*		*Ref*	
Factory worker	2954	1.01 (0.95–1.07)		1.07 (1.00–1.14)	
Administrative or professional	1704	0.83 (0.78–0.89)		0.94 (0.87–1.00)	
Retired	2239	0.99 (0.92–1.06)		1.07 (1.00–1.16)	
Other^d^	2917	0.99 (0.94–1.05)		1.02 (0.96–1.08)	
**Household income, Yuan**					
< 10,000	3725	*Ref*	< 0.001	*Ref*	< 0.01
10,000–19,999	4603	0.94 (0.90–0.99)		0.97 (0.92–1.02)	
≥ 20,000	7224	0.86 (0.82–0.90)		0.93 (0.88–0.98)	
**Household size**					
≤ 2 people	2569	*Ref*	< 0.001	*Ref*	0.10
3 people	4735	0.98 (0.93–1.03)		1.01 (0.96–1.07)	
≥ 4 people	8248	1.01 (0.96–1.06)		1.04 (0.99–1.10)	
**Household fridge, years**					
No fridge	6715	*Ref*	< 0.001	*Ref*	< 0.001
1–9	3786	0.94 (0.90–0.98)		0.97 (0.93–1.02)	
10–19	3655	0.85 (0.81–0.90)		0.91 (0.86–0.96)	
≥ 20	1396	0.79 (0.74–0.85)		0.87 (0.81–0.93)	
**Current smoker**^**e**^					
Yes vs. no	5552	1.02 (0.97–1.07)	0.47	1.02 (0.97–1.07)	0.43
**Alcohol intake**^**f**^					
Abstainer	7192	*Ref*	< 0.001	*Ref*	< 0.001
Occasional intake	4709	0.84 (0.81–0.88)		0.87 (0.83–0.91)	
Ever-regular intake	3651	0.86 (0.81–0.90)		0.89 (0.84–0.93)	
**Physical activity tertile**					
Bottom tertile	4910	*Ref*	0.93	*Ref*	0.80
Middle tertile	5057	0.95 (0.92–1.00)		1.00 (0.95–1.05)	
Top tertile	5585	0.98 (0.94–1.02)		0.99 (0.94–1.05)	
**Regular fruit intake**^**g**^					
Yes vs. no	4284	0.96 (0.93–1.00)	0.08	1.03 (0.99–1.07)	0.15
**BMI groups, kg/m**^**2**^					
Underweight [< 18.50]	718	1.16 (1.07–1.25)	< 0.001	1.11 (1.03–1.20)	< 0.001
Normal [18.5– < 25]	9963	*Ref*		*Ref*	
Overweight [≥ 25– < 30]	4258	0.91 (0.88–0.95)		0.92 (0.89–0.95)	
Obese [≥ 30]	613	0.98 (0.90–1.07)		0.97 (0.89–1.06)	
**Blood transfusion**					
Yes vs. no	759	1.12 (1.04–1.21)	< 0.01	1.11 (1.03–1.19)	< 0.01
**Self-rated health**					
Good	6760	*Ref*	< 0.001	*Ref*	< 0.001
Fair	7079	1.14 (1.10–1.18)		1.12 (1.09–1.16)	
Poor	1713	1.35 (1.27–1.42)		1.29 (1.22–1.37)	

BMI, body mass index; MET, metabolic equivalent of task; NS, not significant.

^a^Adjusted for age in 5-year categories, sex and study site (ten sites) where possible, ^b^Adjusted for age in 5-year categories, sex and study site (ten sites), birth cohort, education, occupation, household income, number of years with a household fridge, alcohol intake, history of blood transfusion, body mass index and self-rated health where possible, ^c^p_trend_ presented for ordered categorical variables, ^d^Other occupation includes house-wife/husband, self-employed, unemployed, other or not-stated, ^e^Smoking: Current smoker includes those reporting occasional or current smoking. ^f^Alcohol: ever-regular includes participants reporting monthly, reduced intake, weekly or ex-regular alcohol intake. ^g^Regular fruit intake includes participants report intake 4 or more times per week.

### Genetic risk factors associated with HBsAg positivity

Of the 68,899 participants included in the genetic analysis, 2069 (3.0%) participants were HBsAg positive (Supplementary Table 6). Among the 18 SNPs studied, the risk allele frequency (RAF) varied by study site (Supplementary Table 7), with up to a twofold difference for certain SNPs (e.g. rs652888 G allele RAF 0.16 in Henan and 0.31 in Liuzhou). Overall 17 SNPs were associated with higher odds of HBsAg positivity, with 13 passing the significance threshold after multiple-testing adjustment (Table 3). The strongest associations were observed at SNPs located in the *HLA-DPB1* gene, including rs9277535 (1.48, 1.39–1.58), rs7770370 (1.38, 1.29–1.47) and rs3077 (1.34, 1.25–1.44). Variants rs3130542 and rs2853953 near the *HLA-C* gene were also associated with HBsAg positivity. Non-classical HLA variants associated with HBsAg positivity included rs652888 in *EHMT2* (1.18, 1.10–1.27); rs422951 in *NOTCH4* (1.19, 1.10–1.29) and rs12614 in *CFB* (1.48, 1.26–1.73). Two SNPs in a non-HLA locus were associated with HBsAg positivity: rs1883832 near gene *CD40* (1.12, 1.05–1.19), and rs4821116 near *UBE2L3* (1.10, 1.03–1.17). SNP rs7000921 did not replicate, although the association was directionally consistent with the previous GWAS.

**Table 3:** Genetic variant associations of select SNPs with HBsAg positivity and chronic liver disease^a^.

SNP	Location^b^	Nearest Gene	Risk/other allele	Hepatitis B surface antigen (HBsAg) positivity				Chronic liver disease			
				RAF cases (N = 2069)	RAF controls (N = 67,830)	Odds ratio (95% CI)	*p*-value	RAF cases (N = 1600)	RAF controls (N = 69,437)	Odds ratio (95% CI)	p-value
rs3077	6:33,033,022	*HLA-DPA1*	G/A	0.72	0.65	1.34 (1.25–1.44)	9.72 × 10^–17^	0.70	0.65	1.25 (1.16–1.36)	1.09 × 10^–8^
rs9366816	6:33,104,175	*HLA-DPA3*	C/T	0.53	0.47	1.27 (1.19–1.35)	1.06 × 10^–13^	0.52	0.47	1.20 (1.12–1.29)	4.42 × 10^–7^
rs9277535	6:33,054,861	*HLA-DPB1*	G/A	0.65	0.55	1.48 (1.39–1.58)	5.42 × 10^–32^	0.62	0.56	1.28 (1.19–1.38)	4.53 × 10^–11^
rs7770370	6:33,048,921	*HLA-DPB1*	G/A	0.59	0.51	1.38 (1.29–1.47)	3.42 × 10^–23^	0.57	0.51	1.23 (1.14–1.32)	1.70 × 10^–8^
rs7453920	6:32,730,012	*HLA-DQB2*	G/A	0.92	0.89	1.50 (1.34–1.68)	4.38 × 10^–12^	0.90	0.88	1.26 (1.11–1.42)	1.87 × 10^–4^
rs2856718	6:32,670,255	*HLA-DQ*	T/C	0.59	0.55	1.17 (1.10–1.25)	7.17 × 10^–7^	0.58	0.55	1.11(1.03–1.19)	5.00 × 10^–3^
rs9276370	6:32,707,295	*HLA-DQ*	T/G	0.89	0.85	1.37 (1.24–1.51)	8.56 × 10^–10^	0.88	0.85	1.23 (1.11–1.37)	1.51 × 10^–4^
rs378352	6:32,974,934	*HLA-DOA*	A/G	0.43	0.39	1.17 (1.09–1.24)	1.83 × 10^–6^	0.43	0.39	1.17 (1.09–1.26)	1.66 × 10^–5^
rs3130542	6:31,232,111	*HLA-C*	A/G	0.18	0.17	1.09 (1.00–1.18)	4.95 × 10^–2^	0.16	0.17	0.95 (0.87–1.05)	3.41 × 10^–1^
rs2853953	6:31,235,505	*HLA-C*	G/A	0.90	0.92	1.14 (1.01–1.28)	2.87 × 10^–2^	0.92	0.90	1.11 (0.98–1.27)	1.07 × 10^–1^
rs652888	6:31,851,234	*EHMT2*	G/A	0.27	0.23	1.18 (1.10–1.27)	3.84 × 10^–6^	0.26	0.23	1.11 (1.03–1.21)	9.41 × 10^–3^
rs1419881	6:31,130,593	*TCF19*	A/G	0.57	0.55	1.09 (1.02–1.16)	7.62 × 10^–3^	0.59	0.55	1.16 (1.08–1.24)	6.30 × 10^–5^
rs422951	6:32,188,383	*NOTCH4*	T/C	0.82	0.79	1.19 (1.10–1.29)	3.27 × 10^–5^	0.82	0.79	1.15 (1.05–1.26)	2.36 × 10^–3^
rs12614	6:31,914,179	*CFB*	C/T	0.96	0.94	1.48 (1.26–1.73)	1.76 × 10^–6^	0.95	0.94	1.15 (0.98–1.35)	8.70 × 10^–2^
rs421446	6:33,174,783	*MIR219A1*	G/A	0.68	0.62	1.28 (1.20–1.37)	6.21 × 10^–13^	0.66	0.62	1.15 (1.07–1.24)	2.79 × 10^–4^
rs4821116	22:21,973,319	*UBE2L3*	C/G	0.63	0.62	1.10 (1.03–1.17)	6.09 × 10^–3^	0.64	0.62	1.12 (1.04–1.21)	2.53 × 10^–3^
rs1883832	20:44,746,982	*CD40*	T/C	0.43	0.39	1.12 (1.05–1.19)	7.86 × 10^–4^	0.41	0.39	1.09 (1.01–1.17)	2.36 × 10^–2^
rs7000921	8:20,393,206	*INTS10*	T/C	0.74	0.74	1.03 (0.96–1.10)	4.49 × 10^–1^	0.75	0.74	1.05 (0.97–1.14)	2.34 × 10^–1^

HBsAg, hepatitis B surface antigen; SNP, single nucleotide polymorphism; RAF, risk allele frequency.

^a^Chronic liver disease includes prevalent liver disease (chronic viral hepatitis B, cirrhosis or liver cancer) and incident liver disease (liver cancer C22, Liver cirrhosis (including: alcoholic liver disease, fibrosis and cirrhosis of liver) (ICD-10: K70, K74), hepatic failure K72 [hepatitis failure, not elsewhere classified); ^b^Location as per Build 37 (GRCh37.p13).

Overall 14 of 18 SNPs tested were associated with CLD (Table 3), with 11 having p values < 0.003. The SNPs with the strongest associations included HLA variants rs9277535 (1.28, 1.19–1.38), rs7770370 (1.23 1.14–1.32) and rs3077 (1.25, 1.16–1.36). SNPs rs9266816 near *HLA-DPA3* (1.20, 1.12–1.29), rs7453920 near *HLA-DQB2* (1.26, 1.11–1.42) and rs9276370 (1.23, 1.11–1.37) were also associated with CLD, as were non-classical HLA variants 1419881 near *TCF19* (1.16, 1.08–1.24) and rs421446 near *MIR219A1* (1.15, 1.07–1.24).

Among the 2069 HBsAg positive participants, rs652888 was the only SNP showing an association with CLD (n cases = 406; 1.73, 1.12–2.64) (Supplementary Table 8). In analyses of anti-HBc positive participants (n = 769 from the subset of 2000), only rs7453920 showed an association with HBsAg positivity (n = 49; 3.22, 1.35–9.62) (Supplementary Table 9).

## Discussion

This large nationwide study of Chinese adults presents findings on both non-genetic and genetic risk factors associated with chronic HBV infection. While HBsAg prevalence was 3% in the overall cohort, this varied greatly by study site, with younger age, male sex, socioeconomic factors, alcohol intake and BMI strongly correlated with HBsAg positivity. We also replicated findings of existing GWAS for genetic variants previously associated with chronic HBV, and showed that several of these were additionally associated with risk of CLD. This is the largest population-based study in China to assess both non-genetic and genetic risk factors associated with chronic hepatitis B infection, highlighting the role that numerous risk factors may play in chronic hepatitis B infection.

Several large nationwide surveys in China have previously reported regional variation in HBsAg prevalence. Historically rates of chronic HBV have been higher in rural, western regions of China, but with widespread urbanization and mass migration of rural workers to large coastal cities and eastern provinces, these patterns have been shifting^26^. A nationally representative serosurvey conducted in 2006^27^ of ≈ 41,000 people aged 1–59 years recruited from 31 provinces measured HBsAg in serum blood samples using ELISA, reported higher HBsAg prevalence in western (8.3%) and rural (7.3%), compared to eastern (6.5%) and urban (6.8%) areas. However, there was large variation within these broad geographical regions—for example in Western China, HBsAg prevalence was 11.6%^28^ and 3.9%^29^ in Sichuan and Gansu province respectively. A more recent study in 2 million men aged 21–49 years in rural China enrolled in the National Free Preconception Health Examination project (NFPHEP) between 2010 and 2012 reported HBsAg prevalence of 7.7%, 5.5% and 6.5% in Eastern, Central and Western China respectively^30^, while other large population based cross-sectional studies, mostly conducted in Eastern China, have reported higher HBsAg prevalence among areas of lower socioeconomic status^31^, coastal areas^32^ and areas containing a higher proportion of immigrants^33^. In CKB, we also found large geographic variation in HBsAg prevalence, where study sites in southern and eastern China had higher HBsAg prevalence than western and north-eastern sites, and urban sites tended to have higher prevalence than rural sites. This regional variation in HBsAg prevalence highlights both the need to draw on populations from diverse areas of China, where the relative importance of correlates with chronic HBV may vary, and that pooled estimates across large regions may obscure important intra-region differences in HBsAg prevalence. The lower prevalence of HBsAg positivity in CKB compared to the 2006 National serosurvey^27^ which reported an overall HBsAg prevalence of 7.2% (30–60 years: 8.6%) may reflect this regional variation in HBsAg prevalence, in addition to the CKB cohort including adults aged over 59 years (with lower HBsAg prevalence), and the HBsAg test used in CKB having lower sensitivity than ELISA^34^.

The trend of HBsAg positivity in relation to age has shifted over recent decades; as the proportion of vaccinated younger adults increases, HBsAg prevalence peaks at older ages. For example, the 2006 National serosurvey^27^ reported peak HBsAg prevalence in 20–29 years olds (10.5%), while another large cross-sectional study of ≈ 87,000 adults recruited in 2009–2010 in Eastern China reported HBsAg peaked in participants aged 35–40 years^31^ at 11.6%, and a third population based study in 2013 in Western China reported peak prevalence in 53–57 year olds at 10.5%^35^. Prevalence tends to decrease with age beyond this peak, as more of the population undergoes HBsAg seroclearance, and a proportion of the infected individuals are diagnosed and treated, or die from liver related disease. The inverse association observed between older age and HBsAg prevalence is consistent with this, as participants in CKB are from the pre-vaccine era and thus largely unvaccinated.

The higher levels of HBsAg positivity among men compared with women has been described in past studies, including an absolute difference of 3% in the 2006 National serosurvey^27^ (8.6% men; 5.7% women) and up to a twofold relative difference in odds of HBsAg positivity in other large population based studies^36–38^. This is similar to our finding of a 1.4 fold greater risk in HBsAg positivity in men than in women. This sex disparity is hypothesized to be related to a differential HBV-related immune response where immune clearance of serum HBsAg is achieved in a higher proportion of women than men, in addition to women gaining better protection from HBV vaccination^39^.

Past studies in China have also reported on the association between education level and HBsAg positivity^27,36,40–42^, with most showing an inverse association, consistent with our findings. Findings for occupation have been mixed, although agricultural work has been associated with HBsAg positivity in several past studies^27,40,43^, consistent with the higher prevalence of HBsAg positivity in agricultural workers in our study, which may reflect geographic variation and socioeconomic status. Two past studies in Henan^37^ and Jilin^44^ also reported no association between smoking and HBsAg positivity, while few studies have examined the association between self-rated health, alcohol intake or BMI and HBsAg positivity. Self-rated health is likely a marker of socioeconomic status, consistent with higher HBsAg prevalence among participants with lower levels of education described in past studies. Two existing studies reporting the association between HBsAg and BMI had conflicting findings—one population based study of ≈ 400,000 adults in Sichuan province found participants with BMI ≥ 25 kg/m^2^ were significantly more likely to be HBsAg positive compared to normal weight (BMI 18.5–25 kg/m^2^) counterparts (OR 1.08, 95% CI 1.05–1.11)^44^; while the other reported^45^ in ≈ 3500 adults in Shanghai, an inverse association with odds of HBsAg positivity and BMI, where participants with BMI ≥ 28 kg/m^2^ were 49% (95% CI 6–72%) less likely to be HBsAg positive than participants of normal weight. The association we observed between HBsAg positivity and BMI is consistent with this latter study, and may reflect socioeconomic status or reverse causation, whereby participants with chronic HBV may have lost weight in the course of their illness. Furthermore, a U-shaped association between BMI and cirrhosis in CKB has been previously described^46^. Two past studies on Chinese adults in conducted in Sichuan and Guangdong province, reported lower risk of HBsAg positivity among occasional or low to moderate alcohol drinkers compared to never drinkers^35,47^, while another study^32^ conducted in Zhejiang province found that any drinking was associated with a 30% (27–34%) higher risk of HBsAg positivity compared to no drinking. The apparent protective association between alcohol intake and HBsAg positivity in our study may reflect altered behaviour related to alcohol intake among known HBV positive people or people with CLD, for whom abstaining from alcohol may be recommended.

Since the first GWAS on chronic HBV was conducted in 2009, the number of SNPs significantly associated with chronic HBV has expanded from SNPs at HLA class II loci, to include those at HLA class I loci, non-classical HLA SNPs and non-HLA SNPs. Most previous GWAS were based on diagnosed clinical conditions such as CLD or liver cancer^10,12–14,16,22^, meaning that participants with different HBV phenotypes such as less severe disease, or chronic HBV without progression to liver disease, may be under-represented. Furthermore, although most GWAS have been performed in participants of East-Asian ancestry, several used populations from particular geographic areas, and tended to be modest in sample size, ranging from between ≈ 4000^16^ to ≈ 15,000^23^ people. Our study included > 65,000 participants and replicated the associations of 17 SNPs with HBsAg positivity. We did not replicate rs7000921 (*INTS10*) previously reported in a Chinese ancestry case–control study of ≈ 9500 people^24^. However, the phenotype examined in that study was HBsAg positivity among anti-HBc positive individuals, which we had limited power to explore due to the small size of the sub-cohort with anti-HBc data.

Existing evidence suggests there is little overlap between SNPs associated with HBsAg positivity and those associated with progression to HBV-related liver disease, where a systematic review of published SNPs associated with different HBV phenotypes found that the overlap occurred between SNPs associated with HBV positivity and HBV vaccine response, rather than with disease progression^11^. Most past GWAS on disease progression have reported HCC progression among HBsAg positive participants. These differences in population and phenotype reported in past GWAS may explain our finding of 14 of 18 SNPs being associated with CLD: we examined CLD more broadly among all participants regardless of HBsAg status, with limited power to investigate progression to CLD among HBsAg positive participants.

The strengths of this study include its large size from diverse geographic areas, both middle- and older-aged population and breadth of information that enabled investigation of a wide range of both conventional and genetic factors associated with HBsAg positivity. To date risk factors associated with chronic HBV have been focused on factors related to mother to child transmission and age of infection; while evidence around associations of socioeconomic, behavioural and medical factors with chronic HBV among adults is lacking. Given that the key burden of chronic HBV related disease occurs in middle-aged and older adults, and the low diagnosis rate of chronic HBV in China, our study findings help fill the evidence gap. However, our study also has several limitations. First, the RDT HBsAg test has lower sensitivity than laboratory-based tests such as ELISA used in most existing smaller studies^34^, leading to a likely underestimation of HBsAg prevalence, which may be more pronounced in those with lower viral load, such as older participants. Second, due to lack of other hepatitis data (e.g. anti-HBc, e-antigen) in the whole cohort, we were only able to compare HBsAg positive to HBsAg negative individuals. We therefore were unable to detect individuals with occult infection, or investigate other phenotypes of interest such as HBsAg viral clearance. Although this approach is consistent with the approach taken by past GWAS^12,14,22^, several others were able to investigate HBsAg clearance among a cohort of exposed participants^13,23,24^. Third, we investigated SNPs identified in previous GWAS in different populations, mainly from the HLA region, but did not explore further the likely multiple independent effects from various SNPs in this part of the genome, which may vary among different populations. However, our work nonetheless adds to the evidence regarding the likely association between HLA variance and HBsAg positivity in Chinese populations. Four, we did not have information on other relevant risk factors, including drug use, number of sexual partners and vaccination status, in addition to information on viral subtype, which is an important source of disease heterogeneity. Finally, this is a cross-sectional study investigating associations between a range of non-genetic and genetic factors with prevalent chronic HBV, which does not capture risk of incident infection.

In summary, this study adds to the current knowledge of factors associated with HBV chronicity in adults, which may help to inform targeted HBsAg screening, enabling improved diagnosis and capturing of individuals on the HBV care continuum. Future research combining conventional and genetic risk factors, including viral genotypes, could further improve knowledge about the risk of HBV chronicity and disease progression.

## Supplementary Information

Supplementary Information.

## Data Availability

The China Kadoorie Biobank (CKB) is a global resource for the investigation of lifestyle, environmental, blood biochemical and genetic factors as determinants of common diseases. The CKB study group is committed to making the cohort data available to the scientific community in China, the UK and worldwide to advance knowledge about the causes, prevention and treatment of disease. For detailed information on what data is currently available to open access users and how to apply for it, visit: http://www.ckbiobank.org/site/Data+Access. Researchers who are interested in obtaining the raw data from the China Kadoorie Biobank study that underlines this paper should contact ckbaccess@ndph.ox.ac.uk. A research proposal will be requested to ensure that any analysis is performed by bona fide researchers and—where data is not currently available to open access researchers—is restricted to the topic covered in this paper.
