# Supplementary material for: Conventional and genetic risk factors for chronic Hepatitis B virus infection in a community-based study of 0.5 million Chinese adults
Source: Sci Rep. 2022 Jul 15;12:12075. doi: 10.1038/s41598-022-16360-7 (PMC9287541; doi:10.1038/s41598-022-16360-7)
Supplement: Supplementary file 1 — Supplementary Information. [file 41598_2022_16360_MOESM1_ESM.docx]

**Supplementary Materials**

**Conventional and genetic risk factors for chronic Hepatitis B virus infection in a community-based study of 0.5 million Chinese adults**

**Scientific Reports**

Elizabeth Hamilton^1^, Ling Yang^1,2^, Alexander Mentzer^3^; Yu Guo ^4^, Yiping Chen^1,2^, Jun Lv^5^, Robert Fletcher^6^, Neil Wright^1^, Kuang Lin^1^, Robin Walters^1,2^, Christiana Kartsonaki^1,2^, Yingcai Yang^7^, Sushila Burgess^1^, Sam Sansome^1^, Liming Li^1,8^, Iona Y. Millwood^1,2^, Zhengming Chen^1^

1. Clinical Trial Service Unit & Epidemiological Studies Unit (CTSU), Nuffield Department of Population Health, University of Oxford, UK
2. Medical Research Council Population Health Research Unit (MRC PHRU), Nuffield Department of Population Health, University of Oxford, UK
3. The Wellcome Centre for Human Genetics, University of Oxford, UK
4. Chinese Academy of Medical Sciences, Beijing, China
5. Department of Epidemiology and Biostatistics, School of Public Health, Peking University Health Science Center, Beijing, China
6. The George Institute for Global Health, Sydney, Australia
7. NCDs Prevention and Control Department, Shinan CDC, Qingdao, Shandong, China
8. Peking University Center for Public Health and Epidemic Preparedness & Response, Beijing, China

**Address for correspondence:**

Dr Ling Yang

CTSU, Nuffield Department of Population Health

BDI Building, Old Road Campus

University of Oxford

Oxford OX3 7LF, UK

Email: ling.yang@ndph.ox.ac.uk

Dr Iona Millwood

CTSU, Nuffield Department of Population Health

BDI Building, Old Road Campus

University of Oxford

Oxford OX3 7LF, UK

Email: iona.millwood@ndph.ox.ac.uk

Table of Contents

[**Supplementary Table S1. Characteristics of existing GWAS studies on chronic hepatitis B infection^a^** 3](#_Toc98750093)

[**Supplementary Table S2. Single nucleotide polymorphisms significantly associated with chronic hepatitis B infection in existing GWAS^a^** 7](#_Toc98750094)

[**Supplementary Table S4. Change in model fit for sequential addition of each variable to the model, given all previous variables in the multivariable model** 10](#_Toc98750095)

[**Supplementary Table S5. anti-HBc and anti-HBe prevalence by age, sex and study-site^a^** 11](#_Toc98750096)

[**Supplementary Table S6. Baseline characteristics of genetic cohort with HBsAg data** 12](#_Toc98750097)

[**Supplementary Table S7. Risk allele frequency for candidate SNPs by study site** 13](#_Toc98750098)

[**Supplementary Table S8. Genetic variant associations for progression to chronic liver disease among 2,069 HBsAg positive participants** 14](#_Toc98750099)

[**Supplementary Table S9. Genetic variant associations for HBsAg persistence among 769 anti-HBc positive participants** 15](#_Toc98750100)

[**Supplementary Figure S1. Flow chart for inclusion in genetic analyses** 16](#_Toc98750101)

[**Supplementary Figure S2. HBsAg prevalence among select baseline characteristics.** 17](#_Toc98750102)

[**Supplementary Methods S1. Selection of covariates in multivariable model** 18](#_Toc98750103)

### **Supplementary Table S1. Characteristics of existing GWAS studies on chronic hepatitis B infection^a^**

| **Study** | **Ethnicity** | **Study type** | **HBV phenotype**^b^ | **Number of participants^c^** | **Recruitment approach** | **Participant characteristics** | | **Meta-analysis or replication** |
| --- | --- | --- | --- | --- | --- | --- | --- | --- |
|  |  |  |  |  |  | Cases | Controls |  |
| **Chang 2014** | Han Taiwanese | Case-control | HBsAg positivity | Total: 2,688  Cases: 1,623  Controls: 1,064 | Cases: Chang Gung Memorial Hospital, Linkou or Taiwan Liver Cancer Network;  Controls: local residents. | Men aged over 30 years; HBsAg seropositive >6 months. Included men with persistent normal ALT, CHB patients with elevated ALT/HBV DNA and HCC patients. | HBsAg seronegative males with normal liver biochemistry. | Two replication stages and meta-analysis |
| **Hu 2013** | Han Chinese | Case-control | HBsAg clearance | Total: 11,791  Cases: 5,181  Controls:6,610 | Derived from two independent case-control cohorts from Central China (Shanghai) and Southern China (Guangdong).  HBV natural clearance cohort from Jiangsu province randomly selected from population-based screening for HBV. | HBsAg seropositive. In discovery cohort 478/951 participants had HCC. | Naturally cleared  HBV carriers (anti-HBc/anti-HBs positive).  In replication controls are from general population with unknown HBV status. | Two replication stages and meta-analysis |
| **Huang 2020** | East Asian | Cohort | HBsAg clearance | Total: 14,650  Cases: 2,458  Controls: 12,192 | Enrolled from Taiwan Biobank, adults aged 30-70 years in general population. Free from cancer, those HBV vaccination or anti-HCV seropositive excluded. | HBsAg positive and anti-HBc positive. | HBsAg negative and anti-HBc positive. | Replication and HLA imputation |
| **Jiang 2015** | Chinese | Case-control | HBsAg positivity | Total: 18,371  Cases: 9,114  Controls: 9,257 | Recruited from Qidong Liver institute. All subjects were antibody negative for HCV, HIV and had self-reported no other types of liver disease. | HBsAg positive and anti-HBc positive for 6 months. | HBsAg negative, no history of vaccination, CHB or HCC. | Four replication stages and meta-analysis |
| **Kamatani**  **2009** | Japanese | Case-control | HBsAg positivity | Total: 6,387  Cases: 2,086  Controls: 4301 | Case and controls for recruited from Biobank Japan other than: First stage controls– 934 Japanese controls in first stage were volunteers from rotary club; second replication cases –Toranomon Hospital and Hiroshima Liver Study Group hospitals. | CHB based on HBsAg positivity and elevated ALT for at least 6 months. | HBsAg negative, no history of CHB, but positive history of another disease (e.g. cancer, stroke). | Three replication stages and haplotype analysis |
| **Kim 2013** | Korean | Case-control | HBsAg positivity | Total: 4,309  Cases: 2,938  Controls: 1,371 | Patients from outpatient clinic of the Liver Unit at Seoul National University Hospital and Ajou University Medical Centre. Population controls recruited from Korea BioBank.  For replication - cases from Ulsan University Hospital, Seoul Korea and Korean Biobank | HBsAg positive for 6 months. | Details not reported. | Replication and combined-analysis |
| **Li 2016** | Chinese | Case-control | HBsAg clearance | Total: 9,569  Cases: 5,156  Controls: 4,413 | Genotype data derived from several previously published GWAS and in-house data. Naïve controls – randomly selected with no information on HBV infection - unrelated ethnic adult Chinese from Guangxi province | HBsAg and anti-HBc positive for 6 months | HBsAg negative, positive for anti-HBs and anti-HBc | Four replication stages and meta-analysis |
| **Mbarek 2011** | Japanese | Case-control | HBsAg positivity | Total: 9,163  Cases: 2,667  Controls: 6,496 | Biobank Japan for discovery, first and second replication. Third replication participants drawn from hospitals participating in Hiroshima Liver Study Group. | CHB patients (HBsAg seropositive CHB with elevated ALT for more than 6 months) | HBsAg negative, no history of CHB, but positive history of another disease (e.g. cancer, stroke). | Three replication stages and meta-analysis |

^a^US National Human Genome Research Institute Catalog of Published GWAS utilized, in addition to respective studies to complete table^17^, ^b^HBsAg clearance refers to studies comparing HBsAg positive participants (cases) to those who are HBsAg negative but with evidence of HBV exposure (i.e. anti-HBc positive), while HBsAg positivity refers to HBsAg positivity (cases) compared to controls with unknown HBV exposure status (anti-HBc status not reported or known). Combined numbers across discovery and replication cohorts. Abbreviations: GWAS = genome wide association study; HBsAg = hepatitis B surface antigen; HBV = hepatitis B virus; ALT = alanine aminotransferase; CHB = chronic hepatitis B; HCC = hepatocellular carcinoma; anti-HBc = antibody to hepatitis B core antibody; anti-HBs = antibody to hepatitis B surface antigen; HCV = hepatitis C virus; HIV = human immunodeficiency virus.

### **Supplementary Table S2. Single nucleotide polymorphisms significantly associated with chronic hepatitis B infection in existing GWAS^a^**

| **SNP ID** | **Location^b^** | **Nearest gene** | **Discovery** | **Replication** | **Ethnicity** | **EA^c^/Other** | **OR (95 CI%)** | **P-value** |
| --- | --- | --- | --- | --- | --- | --- | --- | --- |
| **rs3077** | 6:33033022 | *HLA-DPA1* | Kamatani 2009 | Kamatani 2009 | Japanese, Thai | **A**/G | 0.56 (0.51-0.61) | 2 x 10^-38^ |
|  |  |  |  | Mbarek 2011 | Thai | **C**/T | 1.87 (1.73-2.01) | 2 x 10^-61^ |
|  |  |  |  | Kim 2013 | Korean | NR | 0.53 (0.48-0.59) | 5 x 10^-39^ |
|  |  |  |  | Jiang 2015 | Chinese | **G**/A | 1.45 (1.39-1.52) | 1 x 10^-53^ |
| **rs9366816** | 6:33104175 | *HLA-DPA3* | Chang 2014 | Chang 2014 | Chinese | **C**/T | 1.43 (1.28-1.60) | 3 x 10^-10^ |
| **rs9277535** | 6:33054861 | *HLA-DPB1* | Kamatani 2009 | Kamatani 2009 | Japanese, Thai | **A**/G | 0.57 (0.51-0.61) | 6 x 10^-39^ |
|  |  |  |  | Kim 2013 | Korean | NR | 0.53 (0.48-0.58) | 4 x 10^-40^ |
|  |  |  |  | Jiang 2015 | Chinese | **G**/A | 1.52 (1.45-1.59) | 1 x 10^-70^ |
|  |  |  |  | Li 2016 | Chinese | **A**/G | 0.69( 0.59-0.81) | 4 x 10^-6^ |
|  |  |  |  | Mbarek 2011 | Thai | **G**/A | 1.77 (1.65-1.91) | 3 x 10^-54^ |
|  |  |  |  | Chang 2014 | Chinese | **G**/A | 1.59 (1.41-1.79) | 5 x 10^-14^ |
| **rs7770370** | 6:33048921 | *HLA-DPB1* | Huang 2020 | Huang 2020 | Chinese | **A**/G | 0.61 (0.56-0.66) | 3 x 10^-35^ |
| **rs7453920** | 6:32730012 | *HLA-DQB2* | Mbarek 2011 | Mbarek 2011 | Japanese | **G**/A | 1.81 (1.62-2.01) | 6 x 10^-28^ |
|  |  |  |  | Hu 2013 | Chinese | **A**/G | 0.53 (0.48-0.59) | 5 x 10^-37^ |
|  |  |  |  | Kim 2013 | Korean | NR | 0.50 (0.44-0.48) | 7 x 10^-26^ |
|  |  |  |  | Chang 2014 | Chinese | **G**/A | 2.31 (1.87-2.85) | 7 x 10^-15^ |
|  |  |  |  | Jiang 2015 | Chinese | **G**/A | 2.00 (1.82-2.17) | 1 x 10^-60^ |
|  |  |  |  | Li 2016 | Chinese | **A**/G | 0.50 (0.36-0.68) | 6 x 10^-6^ |
| **rs2856718** | 6:32670255 | *HLA-DQ* | Mbarek 2011 | Mbarek 2011 | Japanese | **A**/G | 1.56 (1.45-1.67) | 4 x 10^-37^ |
|  |  |  |  | Kim 2013 | Korean | NR | 1.60 (1.46-1.75) | 2 x 10^-24^ |
|  |  |  |  | Jiang 2015 | Chinese | **T**/C | 1.28 (1.22-1.33) | 7 x 10^-28^ |
| **rs9276370** | 6:32707295 | *HLA-DQ* | Chang 2014 | Chang 2014 | Chinese | **T**/G | 1.95 (1.62-2.34) | 2 x 10^-12^ |
| **rs378352** | 6:32974934 | *HLA-DOA* | Jiang 2015 | Jiang 2015 | Chinese | **T**/C | 1.26 (1.20-1.31) | 1 x 10^-23^ |
| **rs3130542** | 6:31232111 | *HLA-C* | Hu 2013 | Hu 2013 | Chinese | **A**/G | 1.33 (1.23-1.44) | 9 x 10^-14^ |
|  |  |  |  | Jiang 2015 | Chinese | **A**/G | 1.17 (1.10-1.24) | 9 x 10^-7^ |
| **rs2853953** | 6:31235505 | *HLA-C* | Jiang 2015 | Jiang 2015 | Chinese | **G**/A | 1.47 (1.35-1.59) | 5 x 10^-20^ |
| **rs652888** | 6:31851234 | *EHMT2* | Kim 2013 | Kim 2013 | Korean | NR | 1.38 (1.22-1.57) | 7 x 10^-13^ |
|  |  |  |  | Jiang 2015 | Chinese | **G**/A | 1.14 (1.08-1.19) | 1 x 10^-6^ |
| **rs1419881** | 6:31130593 | *TCF19* | Kim 2013 | Jiang 2015 | Chinese | **A**/G | 1.12 (1.08-1.18) | 3 x 10^-7^ |
|  |  |  |  | Kim 2013 | Korean | NR | 0.73 (0.66-0.81) | 1 x 10^-18^ |
| **rs422951** | 6:32188383 | *NOTCH4* | Jiang 2015 | Jiang 2015 | Chinese | **A**/G | 1.27 (1.20-1.35) | 5 x 10^-16^ |
| **rs12614** | 6:31914179 | *CFB* | Jiang 2015 | Jiang 2015 | Chinese | **C**/T | 1.89 (1.69-2.08) | 1 x 10^-34^ |
| **rs4821116** | 22:21973319 | *UBE2L3* | Hu 2013 | Hu 2013 | Chinese | **A**/G | 0.82 (0.77-0.87) | 2 x 10^-12^ |
| **rs1883832** | 20:44746982 | *CD40* | Jiang 2015 | Jiang 2015 | Chinese | **T**/C | 1.19 (1.14-1.25) | 3 x 10^-15^ |
| **rs7000921** | 8:20393206 | *INTS10* | Li 2016 | Li 2016 | Chinese | **C**/T | 0.78 (0.73-0.84) | 3 x 10^-12^ |
| **rs421446** | 6:33174783 | *MIR219A1* | Li 2016 | Li 2016 | Chinese | **A**/G | 0.68 (0.58-0.80) | 3 x 10^-6^ |
| **rs28747027^d^** | 6:32654977 | *HLA-DQB1* | Huang 2020 | Huang 2020 | Chinese | **G/**C | 1.41 (1.32–1.52) | 7 x 10^-21^ |
| ^a^US National Human Genome Research Institute Catalog of Published GWAS utilized, in addition to respective studies to complete table, ^b^Build 37 (GRCh37.p13), ^c^Effect allele as reported in the original study. ^d^rs28747027 was not included in our study due to being missing in our hard-call genotype and imputation panel. Abbreviations: GWAS = genome wide association studies; SNP = single nucleotide polymorphism; EA = effect allele as reported in study; OR = odds ratio; NR = not reported. | | | | | | | | |

**Supplementary Table S3. Change in model fit for the addition of each variable to basic model containing age, sex and study site.**

| **Variable** | ***χ*^2^ (Deviance)** | **df** | **p-value** |
| --- | --- | --- | --- |
| Self-rated health | 125.8 | 2 | 4.7 x 10^-28^ |
| Alcohol intake | 68.7 | 2 | 1.2 x 10^-15^ |
| Years with a household fridge | 58.3 | 3 | 1.4 x 10^-12^ |
| Education | 57.2 | 2 | 3.8 x 10^-13^ |
| Occupation | 46.0 | 4 | 2.5 x 10^-9^ |
| Household income | 41.6 | 2 | 9.3 x 10^-10^ |
| BMI category | 40.9 | 3 | 6.9 x 10^-9^ |
| Birth cohort | 13.4 | 3 | 3.8 x 10^-3^ |
| History of blood transfusion | 9.0 | 1 | 2.7 x 10^-3^ |
| Physical activity level^a^ | 4.8 | 2 | 9.1 x 10^-2^ |
| Regular fruit intake^a^ | 3.1 | 1 | 7.7 x 10^-2^ |
| Household size^a^ | 2.7 | 2 | 2.6 x 10^-1^ |
| Current smoker^a^ | 0.5 | 1 | 4.7 x 10^-1^ |

^a^No significant improvement in model fit, and not included in multivariate model. Abbreviations: df = degrees of freedom; BMI = body mass index.

### **Supplementary Table S4. Change in model fit for sequential addition of each variable to the model, given all previous variables in the multivariable model**

| **Variable** | ***χ*^2^ (Deviance)** | **df** | **p-value** |
| --- | --- | --- | --- |
| Age, sex, study site |  |  |  |
| + Self-rated health | 125.8 | 2 | 4.6 x 10^-28^ |
| + Alcohol intake | 60.2 | 2 | 8.4 x 10^-14^ |
| + Education | 43.1 | 3 | 2.4 x 10^-9^ |
| + Years with a household fridge | 29.6 | 3 | 1.7 x 10^-6^ |
| + Occupation | 25.7 | 2 | 2.7 x 10^-6^ |
| + BMI category | 22.0 | 4 | 2.0 x 10^-4^ |
| + Birth cohort | 19.6 | 3 | 2.1 x 10^-4^ |
| + Household income | 7.0 | 2 | 3.0 x 10^-2^ |
| + History of blood transfusion | 6.8 | 1 | 9.3 x 10^-3^ |

Abbreviations: df = degrees of freedom; BMI = body mass index.

### **Supplementary Table S5. anti-HBc and anti-HBe prevalence by age, sex and study-site^a^**

|  | **Overall**  (N=1,986) | **anti-HBc** | | **anti-HBe** | |
| --- | --- | --- | --- | --- | --- |
|  |  | **anti-HBc positive** | **Prevalence (%) (95% CI)** | **anti-HBe positive** | **Prevalence (%) (95% CI)** |
| **Age category** |  |  |  |  |  |
| 30–39 years | 317 | 128 | 41.2 (35.7-46.6) | 128 | 41.5 (36.2-46.9) |
| 40–49 years | 592 | 247 | 41.8 (37.8-45.8) | 246 | 41.4 (37.5-45.4) |
| 50–59 years | 604 | 271 | 45.1 (41.2-49.0) | 271 | 44.9 (41.0-48.9) |
| ≥60 years | 473 | 247 | 50.9 (46.3-55.4) | 245 | 50.4 (45.8-54.9) |
| **Sex** |  |  |  |  |  |
| Men | 761 | 358 | 46.4 (43.1-49.8) | 358 | 46.2 (42.8-49.5) |
| Women | 1,225 | 535 | 44.0 (41.3-46.6) | 532 | 43.6 (40.9-46.2) |
| **Study site** |  |  |  |  |  |
| Qingdao | 198 | 84 | 48.8 (42.1-55.6) | 107 | 47.7 (41.0-54.5) |
| Harbin | 295 | 87 | 34.2 (28.9-39.5) | 85 | 34.9 (29.6-40.2) |
| Haikou | 135 | 91 | 61.7 (53.6-69.8) | 87 | 61.9 (53.7-70.2) |
| Suzhou | 194 | 82 | 44.7 (38.0-51.4) | 94 | 44.8 (38.1-51.5) |
| Liuzhou | 174 | 43 | 52.4 (45.1-59.7) | 84 | 54.4 (47.3-61.5) |
| Sichuan | 162 | 95 | 50.4 (42.5-58.2) | 40 | 51.7 (43.9-59.6) |
| Gansu | 178 | 105 | 25.9 (19.2-32.6) | 94 | 24.1 (17.5-30.7) |
| Henan | 208 | 104 | 46.2 (39.2-53.2) | 100 | 45.6 (38.7-52.6) |
| Zhejiang | 245 | 429 | 41.7 (35.7-47.8) | 104 | 39.7 (33.6-45.7) |
| Hunan | 197 | 464 | 51.6 (44.7-58.4) | 107 | 51.6 (44.8-58.4) |

^a^Standardized by age in 5-year categories, sex and region where appropriate. Abbreviations: anti-HBc = antibody to hepatitis B core antigen; anti-HBe = antibody to hepatitis B e-antigen.

### **Supplementary Table S6. Baseline characteristics of genetic cohort with HBsAg data**

|  | **HBsAg Negative**  (N=67,829) | **HBsAg Positive**  (N=2,069) | **Overall**  (N=69,898) |
| --- | --- | --- | --- |
| **Age (years),** mean (SD) | 52.3 (10.7) | 50.0 (9.92) | 52.2 (10.7) |
| **Male, %** | 40.1 | 44.9 | 40.2 |
| **Rural study site, %** | 54.1 | 46.0 | 53.8 |
| **Married^a^, %** | 90.6 | 91.4 | 90.6 |
| **No formal education, %** | 18.1 | 16.3 | 18.1 |
| **Agricultural occupation, %** | 39.8 | 36.0 | 39.7 |
| **Income ≥ 20,000 Yuan, %** | 40.3 | 41.3 | 40.3 |
| **Household size ≥ 4 people, %** | 49.9 | 52.0 | 50.0 |
| **No household fridge, %** | 44.1 | 43.9 | 44.1 |
| **Current smoker^b^, %** |  |  |  |
| Men | 71.4 | 72.9 | 71.4 |
| Women | 4.3 | 4.2 | 4.2 |
| **Ever-regular alcohol intake^c^, %** |  |  |  |
| Men | 49.6 | 47.7 | 49.6 |
| Women | 4.2 | 4.2 | 4.2 |
| **Dietary factors, regular intake^d^,%** |  |  |  |
| Vegetable intake | 98.6 | 98.7 | 98.6 |
| Fruit intake | 30.0 | 27.6 | 29.9 |
| **Physical activity (MET-hours/day),** mean (SD) | 20.6 (14.0) | 20.7 (14.5) | 20.6 (14.0) |
| **SBP (mmHg),** mean (SD) | 132 (21.4) | 129 (20.1) | 132 (21.4) |
| **BMI (mg/kg^2^),** mean (SD) | 23.8 (3.5) | 23.6 (3.5) | 23.8 (3.5) |
| **Blood transfusion, %** | 4.5 | 5.5 | 4.5 |
| **Poor self-rated health, %** | 10.3 | 11.6 | 10.3 |
| **Prior disease history, %** |  |  |  |
| Chronic HBV/cirrhosis | 0.9 | 12.0 | 1.2 |
| Cancer | 0.5 | 0.7 | 0.5 |
| CHD | 3.7 | 2.1 | 3.6 |
| Stroke or TIA | 1.7 | 1.2 | 1.7 |
| Diabetes | 3.4 | 2.6 | 3.4 |

**^a^**Married: Participants who reported currently being married, **^b^**Smoking: Current smoker includes those reporting occasional or current smoking, **^c^**Alcohol: Ever-regular alcohol intake includes participants reporting monthly, reduced intake, weekly or ex-regular alcohol intake, ^d^Dietary factors: regular includes participants report intake 4 or more times per week. Abbreviations: HBsAg = hepatitis B surface antigen; MET = metabolic equivalent of task; SBP = systolic blood pressure; BMI = body mass index; CHD = coronary heart disease; TIA = transient ischaemic attack

### **Supplementary Table S7. Risk allele frequency for candidate SNPs by study site**

| **SNP** | **RA** | **Qingdao** | **Harbin** | **Haikou** | **Suzhou** | **Liuzhou** | **Sichuan** | **Gansu** | **Henan** | **Zhejiang** | **Hunan** |
| --- | --- | --- | --- | --- | --- | --- | --- | --- | --- | --- | --- |
| rs3077 | G | 0.60 | 0.60 | 0.70 | 0.66 | 0.73 | 0.68 | 0.55 | 0.59 | 0.69 | 0.74 |
| rs9366816 | C | 0.43 | 0.45 | 0.47 | 0.50 | 0.43 | 0.47 | 0.45 | 0.45 | 0.52 | 0.51 |
| rs9277535 | G | 0.46 | 0.49 | 0.64 | 0.56 | 0.64 | 0.63 | 0.49 | 0.47 | 0.59 | 0.67 |
| rs7770370 | G | 0.46 | 0.46 | 0.58 | 0.52 | 0.57 | 0.55 | 0.44 | 0.45 | 0.54 | 0.59 |
| rs7453920 | G | 0.83 | 0.86 | 0.92 | 0.88 | 0.94 | 0.89 | 0.86 | 0.86 | 0.89 | 0.91 |
| rs2856718 | T | 0.53 | 0.53 | 0.54 | 0.52 | 0.60 | 0.57 | 0.53 | 0.53 | 0.54 | 0.62 |
| rs9276370 | T | 0.81 | 0.83 | 0.89 | 0.85 | 0.91 | 0.86 | 0.83 | 0.84 | 0.86 | 0.89 |
| rs378352 | A | 0.32 | 0.35 | 0.46 | 0.40 | 0.43 | 0.41 | 0.32 | 0.36 | 0.41 | 0.47 |
| rs3130542 | A | 0.16 | 0.14 | 0.16 | 0.14 | 0.19 | 0.19 | 0.17 | 0.14 | 0.17 | 0.22 |
| rs2853953 | G | 0.82 | 0.86 | 0.96 | 0.92 | 0.96 | 0.94 | 0.87 | 0.85 | 0.93 | 0.97 |
| rs652888 | G | 0.18 | 0.17 | 0.29 | 0.25 | 0.31 | 0.28 | 0.18 | 0.16 | 0.26 | 0.30 |
| rs1419881 | A | 0.56 | 0.56 | 0.56 | 0.54 | 0.52 | 0.54 | 0.51 | 0.55 | 0.56 | 0.58 |
| rs422951 | T | 0.71 | 0.74 | 0.83 | 0.83 | 0.81 | 0.81 | 0.74 | 0.73 | 0.85 | 0.85 |
| rs12614 | C | 0.90 | 0.92 | 0.98 | 0.95 | 0.97 | 0.94 | 0.91 | 0.91 | 0.96 | 0.97 |
| rs421446 | G | 0.62 | 0.63 | 0.65 | 0.66 | 0.60 | 0.62 | 0.55 | 0.61 | 0.67 | 0.64 |
| rs4821116 | C | 0.67 | 0.67 | 0.54 | 0.61 | 0.53 | 0.60 | 0.67 | 0.66 | 0.60 | 0.58 |
| rs1883832 | T | 0.35 | 0.35 | 0.49 | 0.39 | 0.50 | 0.41 | 0.34 | 0.34 | 0.38 | 0.42 |
| rs7000921 | T | 0.74 | 0.74 | 0.73 | 0.74 | 0.74 | 0.73 | 0.73 | 0.74 | 0.73 | 0.74 |

Abbreviations: SNP = single nucleotide polymorphism; RA = risk allele.

### **Supplementary Table S8. Genetic variant associations for progression to chronic liver disease among 2,069 HBsAg positive participants**

| **SNP** | **Location^a^** | **Nearest Gene** | **Risk /other allele** | **RAF cases**  (N=406) | **RAF controls**  (N=1,663) | **Odds ratio (95% CI)** | **p-value** |
| --- | --- | --- | --- | --- | --- | --- | --- |
| rs3077 | 6:33033022 | *HLA-DPA1* | G/A | 0.70 | 0.65 | 1.02 (0.68-1.55) | 0.93 |
| rs9366816 | 6:33104175 | *HLA-DPA3* | C/T | 0.52 | 0.47 | 0.97 (0.66-1.44) | 0.89 |
| rs9277535 | 6:33054861 | *HLA-DPB1* | G/A | 0.62 | 0.56 | 1.39 (0.93-2.11) | 0.11 |
| rs7770370 | 6:33048921 | *HLA-DPB1* | G/A | 0.57 | 0.51 | 1.22 (0.83-1.79) | 0.32 |
| rs7453920 | 6:32730012 | *HLA-DQB2* | G/A | 0.90 | 0.88 | 1.81 (0.92-4.11) | 0.12 |
| rs2856718 | 6:32670255 | *HLA-DQ* | T/C | 0.58 | 0.55 | 0.96 (0.64-1.42) | 0.82 |
| rs9276370 | 6:32707295 | *HLA-DQ* | T/G | 0.88 | 0.85 | 1.26 (0.71-2.4 ) | 0.46 |
| rs378352 | 6:32974934 | *HLA-DOA* | A/G | 0.43 | 0.39 | 1.04 (0.7-1.54 ) | 0.83 |
| rs3130542 | 6:31232111 | *HLA-C* | A/G | 0.16 | 0.17 | 0.87 (0.5-1.42 ) | 0.59 |
| rs2853953 | 6:31235505 | *HLA-C* | G/A | 0.92 | 0.90 | 1.18 (0.62-2.54) | 0.65 |
| rs652888 | 6:31851234 | *EHMT2* | G/A | 0.26 | 0.23 | 1.73 (1.12-2.64) | 0.01 |
| rs1419881 | 6:31130593 | *TCF19* | A/G | 0.59 | 0.55 | 1.02 (0.69-1.51) | 0.93 |
| rs422951 | 6:32188383 | *NOTCH4* | T/C | 0.82 | 0.79 | 0.90 (0.56-1.49) | 0.67 |
| rs12614 | 6:31914179 | *CFB* | C/T | 0.95 | 0.94 | 0.78 (0.38-1.81) | 0.52 |
| rs421446 | 6:33174783 | *MIR219A1* | G/A | 0.66 | 0.62 | 0.92 (0.62-1.38) | 0.69 |
| rs4821116 | 22:21973319 | *UBE2L3* | C/G | 0.64 | 0.62 | 1.01 (0.68-1.53) | 0.95 |
| rs1883832 | 20:44746982 | *CD40* | T/C | 0.41 | 0.39 | 1.43 (0.96-2.13) | 0.07 |
| rs7000921 | 8:20393206 | *INTS10* | T/C | 0.75 | 0.74 | 1.12 (0.72-1.79) | 0.63 |

^a^Location as per Build 37 (GRCh37.p13). Abbreviations: HBsAg = hepatitis B surface antigen; SNP = single nucleotide polymorphism; RAF = risk allele frequency.

### **Supplementary Table S9. Genetic variant associations for HBsAg persistence among 769 anti-HBc positive participants**

| **SNP** | **Location^a^** | **Nearest Gene** | **Risk /other allele** | **RAF cases**  (N=49) | **RAF controls**  (N=720) | **Odds ratio (95% CI)** | **p-value** |
| --- | --- | --- | --- | --- | --- | --- | --- |
| rs3077 | 6:33033022 | *HLA-DPA1* | G/A | 0.66 | 0.67 | 0.95 (0.61-1.52) | 0.84 |
| rs9366816 | 6:33104175 | *HLA-DPA3* | C/T | 0.49 | 0.48 | 1.02 (0.65-1.59) | 0.93 |
| rs9277535 | 6:33054861 | *HLA-DPB1* | G/A | 0.62 | 0.60 | 1.11 (0.69-1.79) | 0.68 |
| rs7770370 | 6:33048921 | *HLA-DPB1* | G/A | 0.57 | 0.54 | 1.15 (0.73-1.83) | 0.55 |
| rs7453920 | 6:32730012 | *HLA-DQB2* | G/A | 0.95 | 0.88 | 3.22 (1.35-9.62) | 0.02 |
| rs2856718 | 6:32670255 | *HLA-DQ* | T/C | 0.54 | 0.56 | 0.94 (0.60-1.48) | 0.79 |
| rs9276370 | 6:32707295 | *HLA-DQ* | T/G | 0.92 | 0.86 | 2.07 (1.01-4.87) | 0.07 |
| rs378352 | 6:32974934 | *HLA-DOA* | A/G | 0.43 | 0.41 | 1.11 (0.71-1.72) | 0.65 |
| rs3130542 | 6:31232111 | *HLA-C* | A/G | 0.16 | 0.18 | 0.92 (0.50-1.60) | 0.79 |
| rs2853953 | 6:31235505 | *HLA-C* | G/A | 0.93 | 0.92 | 1.22 (0.57-3.06) | 0.64 |
| rs652888 | 6:31851234 | *EHMT2* | G/A | 0.34 | 0.25 | 1.60 (0.97-2.61) | 0.06 |
| rs1419881 | 6:31130593 | *TCF19* | A/G | 0.53 | 0.54 | 0.96 (0.62-1.48) | 0.85 |
| rs422951 | 6:32188383 | *NOTCH4* | T/C | 0.81 | 0.80 | 1.04 (0.62-1.82) | 0.88 |
| rs12614 | 6:31914179 | *CFB* | C/T | 0.95 | 0.94 | 1.61 (0.65-4.93) | 0.35 |
| rs421446 | 6:33174783 | *MIR219A1* | G/A | 0.63 | 0.65 | 0.90 (0.58-1.43) | 0.66 |
| rs4821116 | 22:21973319 | *UBE2L3* | C/G | 0.63 | 0.62 | 0.93 (0.60-1.47) | 0.76 |
| rs1883832 | 20:44746982 | *CD40* | T/C | 0.45 | 0.38 | 1.46 (0.94-2.26) | 0.09 |
| rs7000921 | 8:20393206 | *INTS10* | T/C | 0.74 | 0.72 | 1.08 (0.66-1.80) | 0.77 |

^a^Location as per Build 37 (GRCh37.p13). Abbreviations: HBsAg = hepatitis B surface antigen; SNP = single nucleotide polymorphism; RAF = risk allele frequency

### **Supplementary Figure S1. Flow chart for inclusion in genetic analyses**

All CKB Participants

N=512,726

GWAS sample

N=100,640

Non-randomly selected

N=24,658

GWAS Randomly selected sample

N=75,982

GWAS Randomly selected sample (CLD analyses)

N=71,037

Regional population outliers^a^

N=4,945

GWAS with HBsAg data

N=69,898

Missing HBsAg

N=1,139

Abbreviations: CKB = China Kadoorie Biobank; GWAS = genome wide association study; HBsAg = Hepatitis B surface antigen; CLD = chronic liver disease

^a^Regional population outliers included those identified by regional principal component analysis of genomic data

### **Supplementary Figure S2. HBsAg prevalence among select baseline characteristics.**

Hepatitis B surface antigen (HBsAg) prevalences displayed (95% CI) are standardized by age (10-year categories) and study site (ten sites) stratified by sex among a) education level, b) household income, c) occupation, d) years with household fridge, e) self-rated health and f) body mass index category (kg/m^2^: underweight<18.5, normal 18.5–<25; overweight ≥25–<30 and obese ≥30).


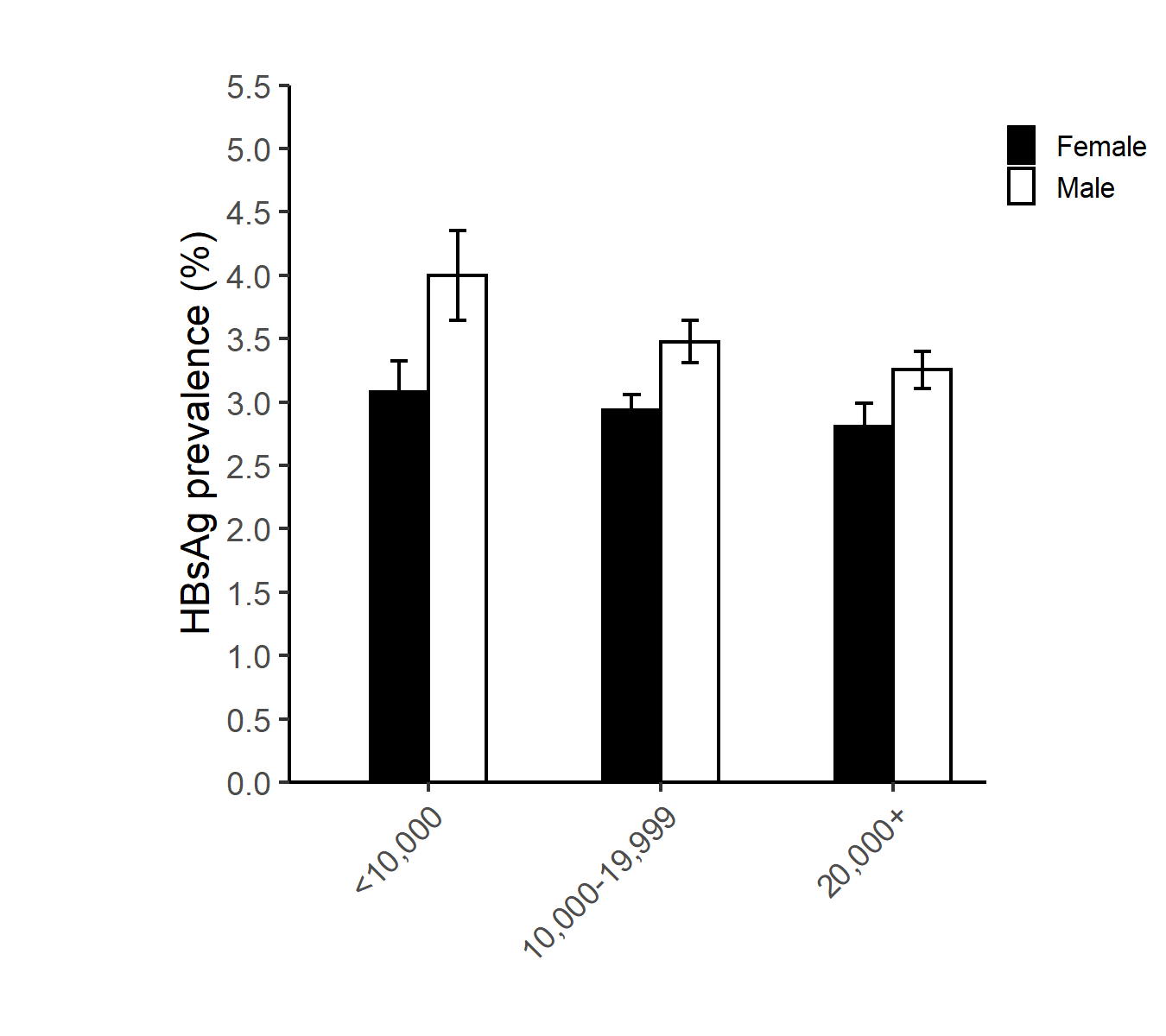

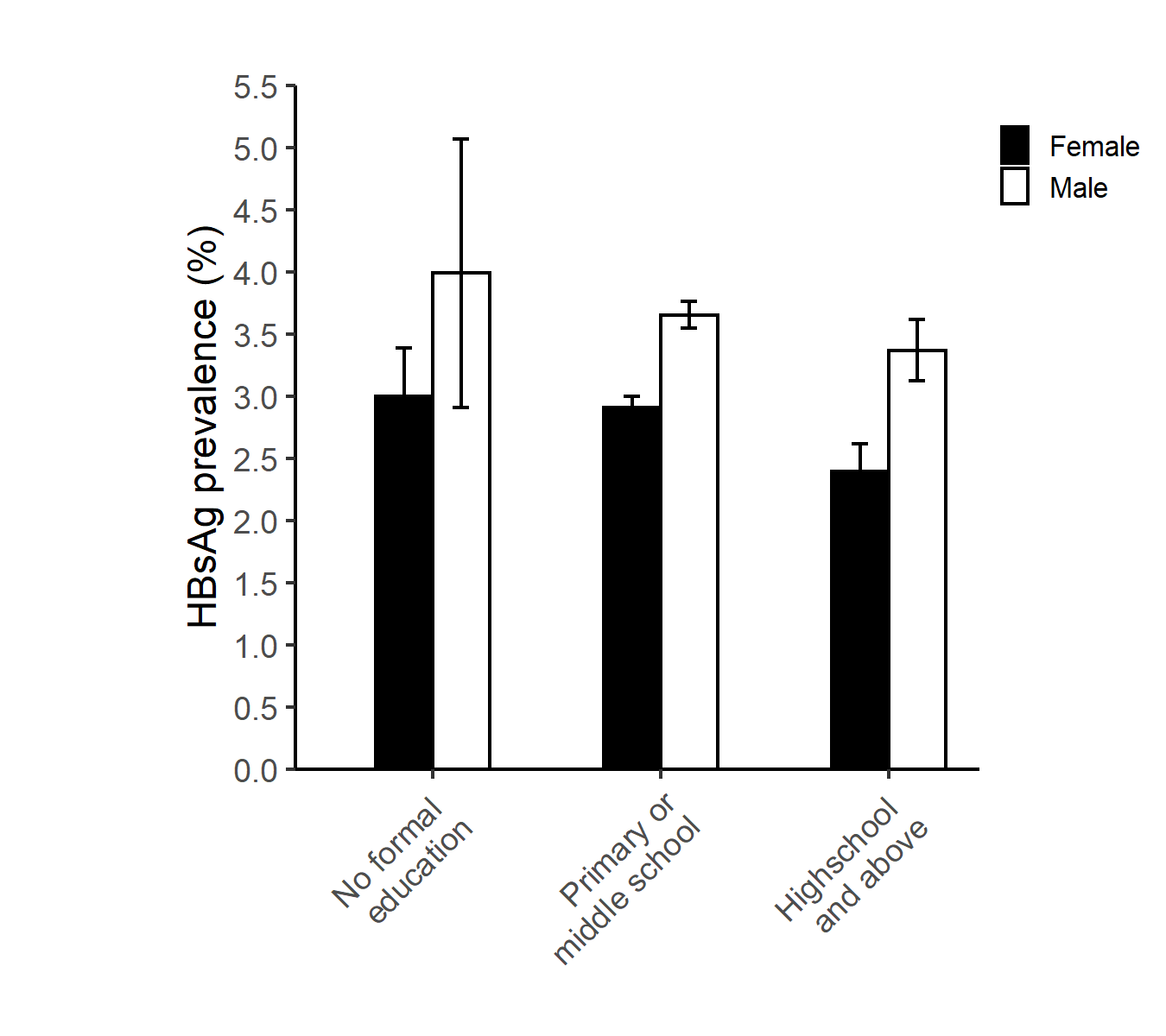


**b)**

**a)**


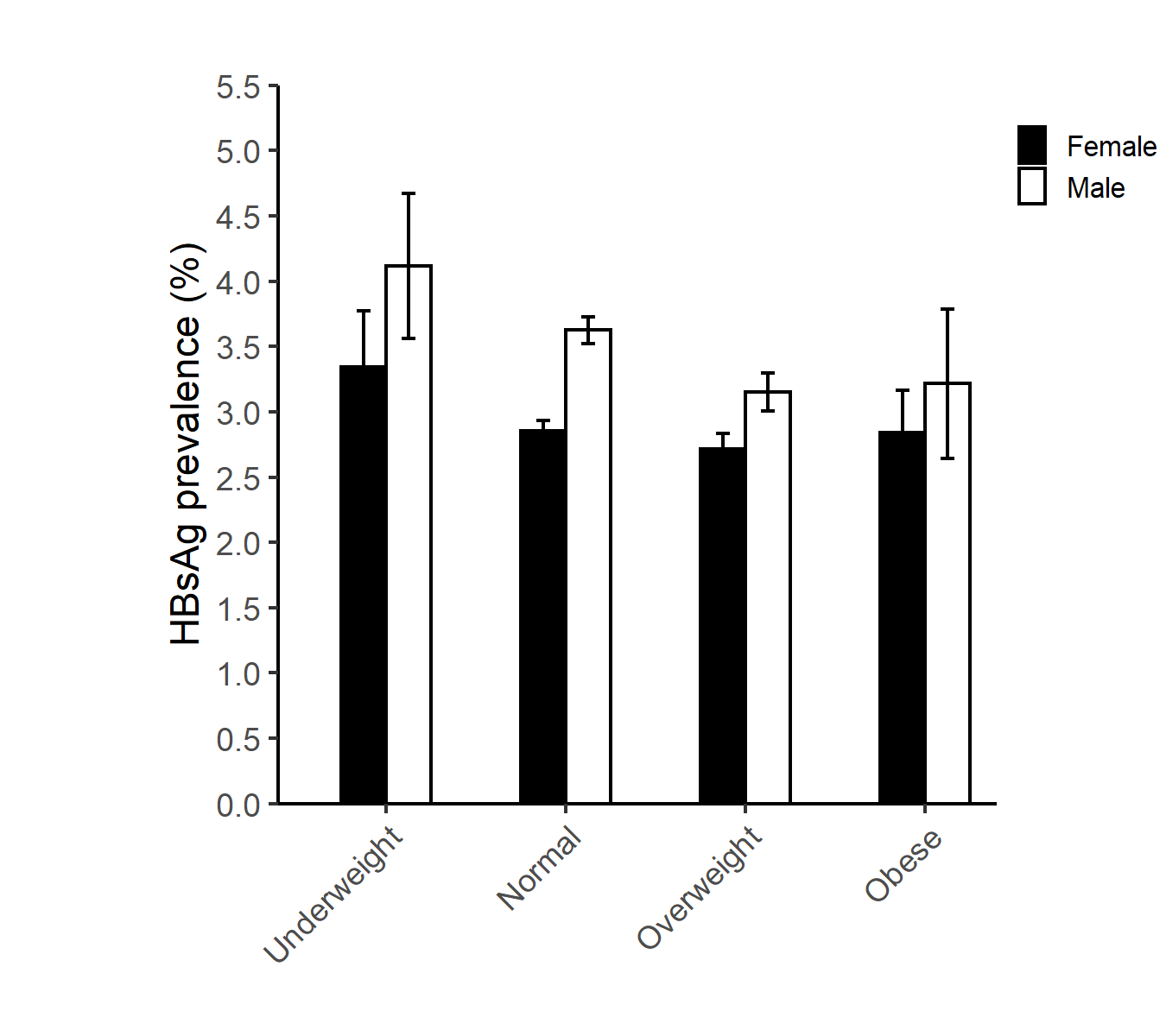

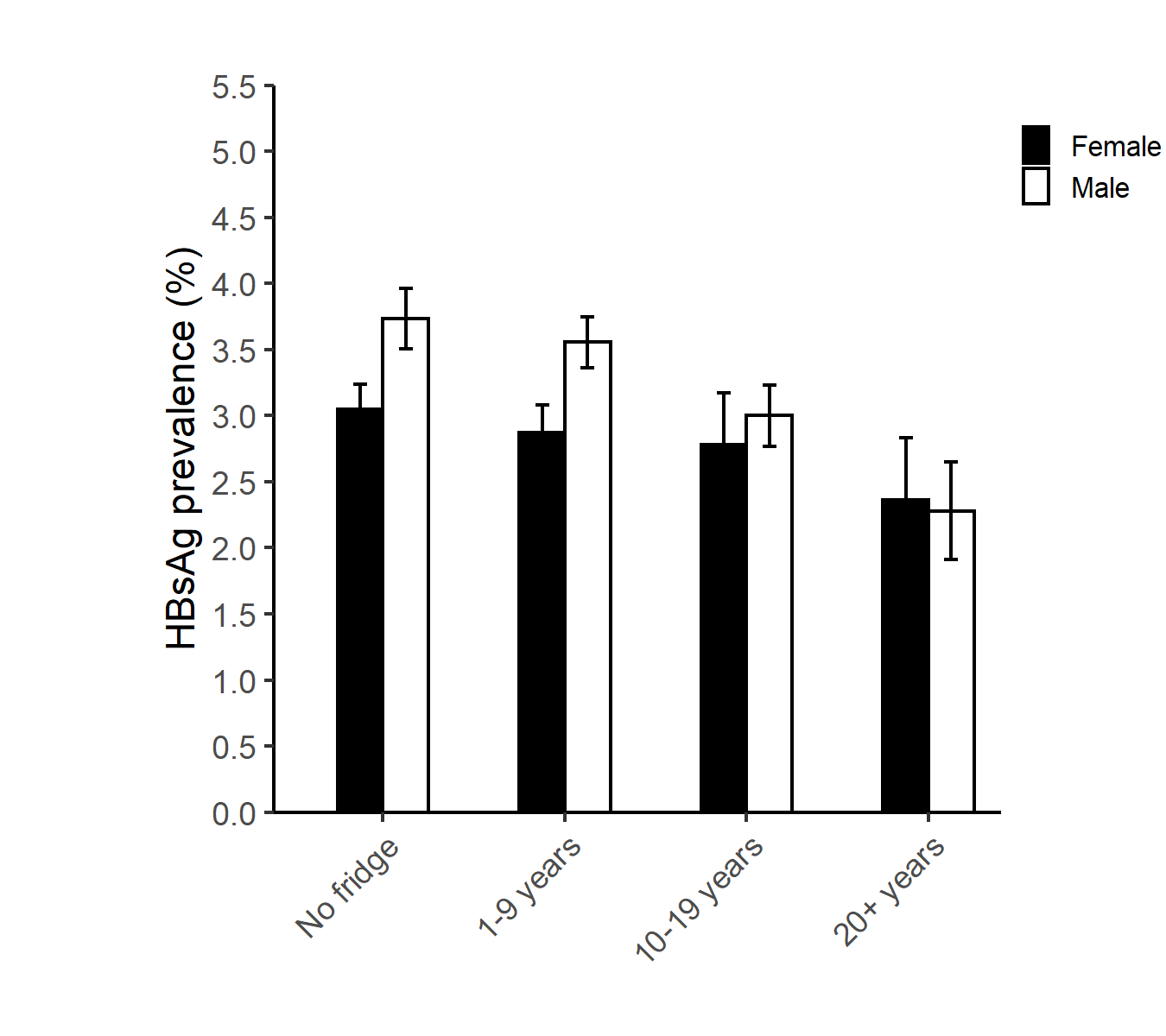

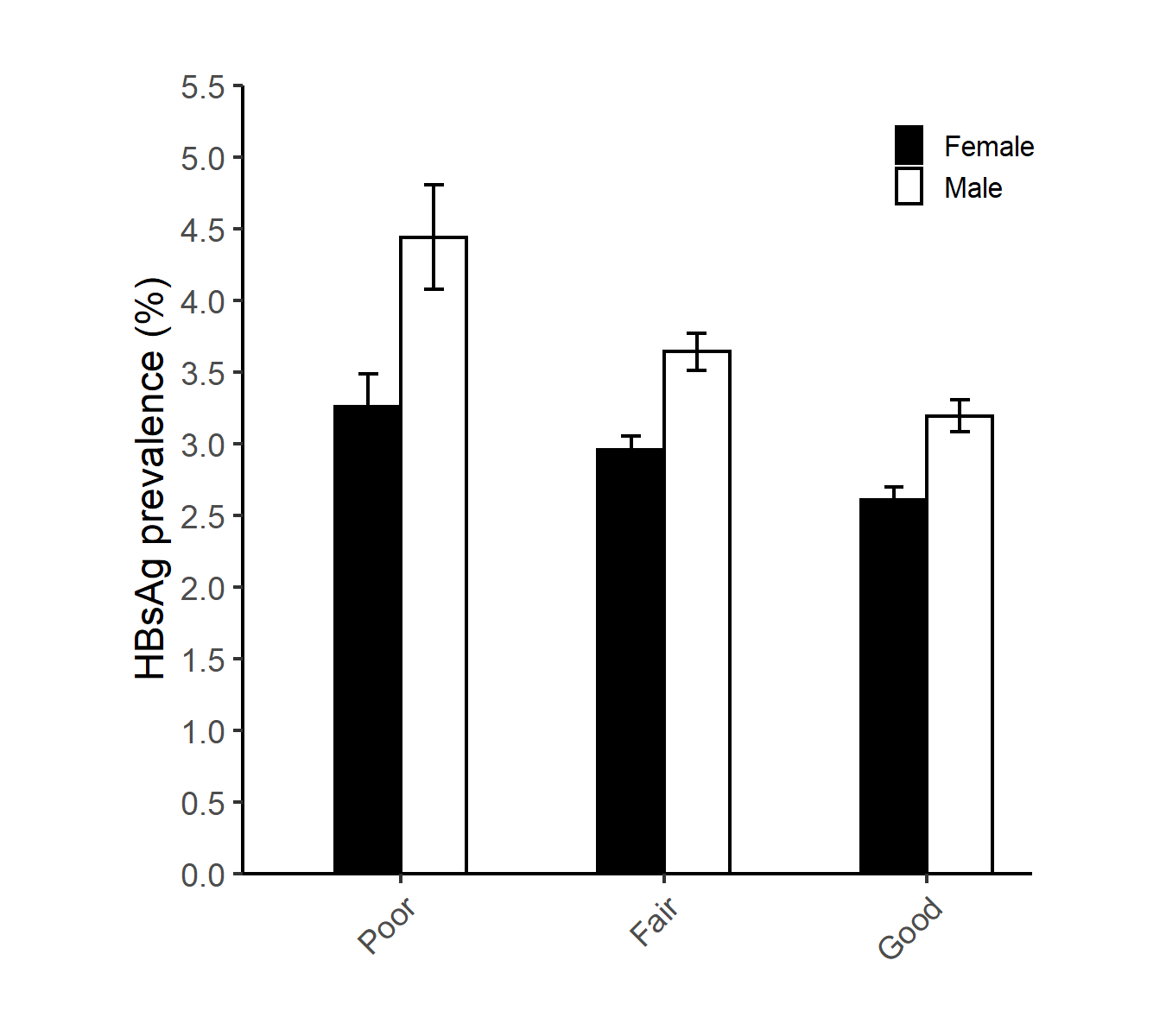

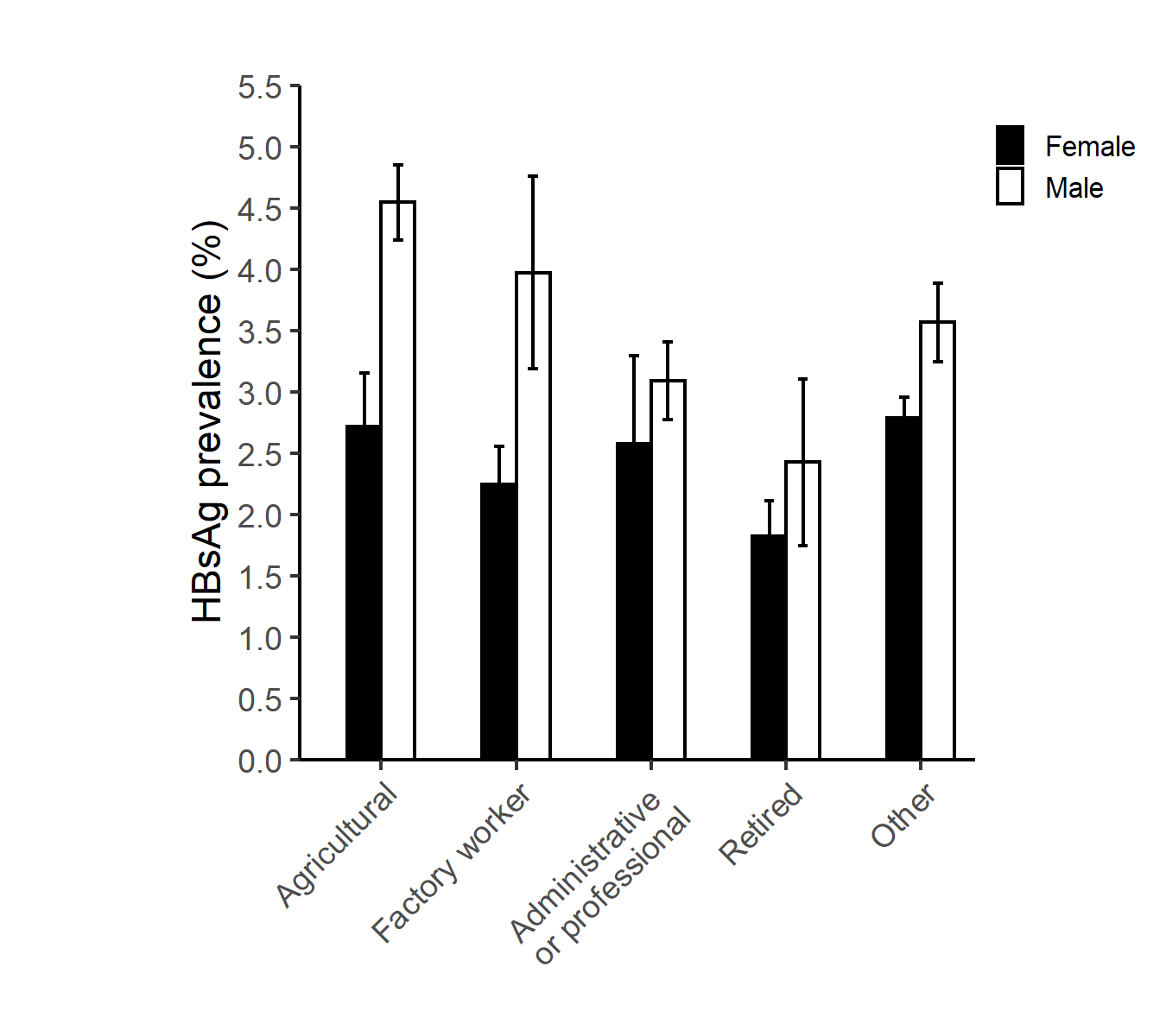


**d)**

**f)**

**e)**

**c)**

### **Supplementary Methods S1. Selection of covariates in multivariable model**

For inclusion of variables in the model a two step forward selection process was used. First, covariates were separately added to the basic model containing age, sex and study site (Supplementary Table S4). Factors that did not significantly improve model fit (physical activity, fruit intake, household size and smoking) were removed. Second, remaining covariates were added to a model containing previous variables, added in order of change in model fit as measured by *χ*^2^**,** where variables that significantly improved model fit were retained. At this stage all variables were retained, leaving the following variables in the multivariable model: age, sex, study site, birth cohort, education, occupation, household income, number of years with a household fridge, alcohol intake, history of blood transfusion, body mass index and self-rated health.
